# Case of Extra-Uterine Fætation

**Published:** 1846-07

**Authors:** M. Bristol, A. S. Sprague

**Affiliations:** Attending Physician, Buffalo; Operating Surgeon, Buffalo


ART. III.—*Case of extra-uterine fœtation. Communicated from Notes furnished by* M. Bristol, M. D., *Attending Physician, and* A. S. Sprague, M. D., *Operating Surgeon.*

Mrs. M. H., aged 32, of thin spare form, had been in delicate health for several years. Had given birth to one child, July, 1836. Although delicate, had been able to attend to her domestic duties until the last year. In Dec., 1845, she was attacked with general symptoms of fever, and continued severely ill for three weeks. On the 19th Jan., 1846, I was again called to prescribe for her. Previously to this, however, her husband had requested my advice at my office, for pain, flatulency, and bloating of the abdomen, for which I prescribed some tonic and carminative. At my visit in Jan., 1846, I found a distinct, hard tumour, with an uniform surface, in the right iliac region, extending over two-thirds of the hypogastric region, attended with some tenderness, and pain toward the right ilium.* Not being satisfied as to its character, I invited Dr. B. Burwell to see the patient. As more of the symptoms agreed with those of ovarian dropsy than any other affection which we at that time suspected, this was the diagnosis determined on in consultation, and she was treated for that disease. The enlargement constantly increased, pain was at times severe over the whole abdominal surface, nights were restless, with occasional sweats, still, she was able to

---

* It is inadvertently omitted to state in the report the interesting fact, that the patient first perceived a tumour in the right iliac region, six years before the attention of Dr. Bristol was called to it. There were no circumstances by which the date of impregnation could be ascertained, apart from the discovery of the tumour.

sit up, and occasionally ride out, in pleasant weather, during the month of March. Her appetite was good.

April 15th,—being called to see her, I found her as large as if in the seventh month of pregnancy, a painful sense of distension being present, difficulty of lying down, frequent desire to urinate, face pale, strength failing, pulse 112.

17th,—in consultation with Dr. B. it was agreed to draw off the fluid; and on the 19th, Dr. B., at my request, punctured the tumour in the linea alba, two inches below the umbilicus. Instead of serum, two pounds of pus escaped through the canula. While the pus was flowing, we discovered a hair in the canula, which was found to be firmly adherent at the extremity within the cavity. Removing the canula the pus ceased to escape; before its removal, however, agonizing pain had come on, which was relieved by large doses of morphine, fomentations, and an enema of starch and laudanum. The nature of the case, as developed by the operation, was now related to the patient, and friends. The danger of an operation to remove the misplaced fœtus, or the parts which still remained, and also, the danger of delay, were fairly represented, and the operation recommended. Dr. Sprague being added to the consultation, coincided in our opinion. On the 19th April, 10 A. M., the operation was performed by Dr. Sprague in the presence of Drs. B. and G. N. Burwell, Drs. G. F. Pratt, H. N. Loomis, and myself. [The remainder of the history of the case is given from notes recorded daily by Dr. Sprague.]

I was desired to see Mrs. H., April 18th, 1846. Found her very feeble, abdomen of the size of a woman advanced to 7 or 8 months in pregnancy, and very tender to the touch. Some long hairs protruded through a wound below the umbilicus, which had been made the day previous in order to discharge the contents of the abdominal tumour. Drs. Bristol, B. Burwell, and myself were of opinion that the case was one of extra-uterine fœtation, and an operation was decided upon for the following morning at 9 o'clock.

19th 9. A. M.—Mrs. H. more comfortable than on yesterday. Abdomen less tender. Pulse 120, and soft. Tinct. opii ℥ii had been administered, *per enema*. at 7, A. M.

Making an incision of four or five inches in length, from the umbilicus toward the pubis, through the linea alba, a full quart of pus was discharged from the peritoneal cavity. I now came in contact with a large cyst containing fluid, Into this I passed a groved needle, and a fluid resembling pus made its appearance. I immediately laid open the cyst from which was discharged not less than three quarts of an oily, purulent matter. I then removed from the cavity a tuft of hairs from five to twelve inches in length, and weighing when dried, ninety nine grains. After the discharge of the matter I made a thorough exploration of the sac, and found attached at the bottom two solid bodies; one about two inches in length, of the size of an ordinary adult finger, formed of bones covered by common integument with hairs interspersed, and two small teeth protruding. The other was an irregular shaped bone, covered in the same manner, with four teeth protruding—two incisions and two molares. The formations seemed to be the result of chance, not of design.

The wound was dressed with three twisted sutures, strips of adhesive plaster, and bandage. Patient complained of pain in her side and shoulder. Pulse 130, sulph. morphiæ gr¼ was directed every hour until relief followed. 1, P. M.—pulse 120.; no abatement of pain; continued morph. 5, P. M.—pulse 112; sleeping comfortably. 9, P, M.—pulse, 115, skin cool and moist; comfortable; omit morphia.

20*th*, 8, *A. M.*—Pulse 115, soft, skin moist, tongue slightly furred, some thirst, bowels somewhat tumid and painful, pains extending to side and shoulder. Fomentations and morphia, if pain continue. 5, P. M.—Pulse 126, hard; no pain, skin moist, bowels not moved since the operation; wound discharges a little, slight nausea, sulph. mag. directed—toast water and gruel for nutriment; anodyne enema after discharge from bowels.

21*st*, 8, *A. M.*—Pulse 112, bowels moved by an enema; has urinated; some tympanites. 5, P. M.—Pulse 128, pain in side, abundant serous discharge from wound; menses appeared, being due at this period.

22*d*, 8, *A. M.*—Pulse 122, no discharge from wound, some cough, menses continue; brandy directed and animal broth. 5, P. M.—Pulse 114, soft, skin moist, tongue cleaning, urinates freely, bowels moved twice, wound discharges freely; morphia and tinct. opii injections have been given pro re nata.

23*d*—Pulse 108 to 126, soft, skin cool and moist; no desire for food; no discharge from wound; about one-third upper part of wound healed by first intention; considerable cough; free spontaneous discharge from bowels. Treatment: laudanum injections, brandy, porter, broths, pro re nata. Poultices have all along been continued.

24*th*—Pulse 110 to 124, soft, skin cool, cough and expectoration; has vomited several times; abdomen less tumid; wound discharges serous fluid freely. Continue treatment: apply sol. chlor. calcis.

25*th*—Pulse 116, soft; skin cool and moist.

*26th*—Pulse 108 to 120, tongue more furred; abdomen more tender; discharge from wound copious and fetid; no desire for food; cough troublesome. Treatment: s. quiniæ, gr. i, every two hours, and other measures as before.

*27th*—Pulse 120, small, symptoms worse. Continue treatment.

*28th*—Pulse 120, feeble; cough and expectoration continue; fetid discharge continues; refuses nourishment and stimulants; constantly failing. From this date, she progressively grew worse, and died on May 2d, 9 A. M.

*Autopsy*, 26 hours after death. Body greatly emaciated; opened abdomen by crucial incision; intense peritoneal inflammation; omentum inflamed and thickened; intestines agglutinated. A small quantity of pus was found diffused among the intestines. Uterus healthy; left ovarium enlarged; right ovarium converted into a large cyst, extending into the left side, capable of containing several quarts of fluid, empty; its walls, adherent to peritoneum and other surrounding parts. Traces of chronic inflammation throughout whole of abdominal cavity.

BUFFALO, June, 1846.

